# Dominant Factors Affecting Regional Inequality of Infant Mortality in Vietnam: A Structural Equation Modelling Analysis

**DOI:** 10.34172/ijhpm.2020.59

**Published:** 2020-04-29

**Authors:** Mai P. Nguyen, Chi M. Nguyen

**Affiliations:** ^1^Department of Medical Services Administration, Ministry of Health, Hanoi, Vietnam.; ^2^Queensland University of Technology, Brisbane, QLD, Australia.; ^3^School of Nursing, Indiana University, Bloomington, IN, USA.

**Keywords:** Infant Mortality, Regional Inequality, Skill Birth Attendance, Socio-Economic Status, Vietnam

## Abstract

**Background:** Despite Vietnam’s acclaiming achievements of reducing overall infant mortality rate (IMR), the IMR decline does not occur equally in all regions in Vietnam. This study aims to investigate dominant factors that affect the inequality of infant mortality across regions in Vietnam during the period 2005-2015.

**Methods:** We use nationally representative data to construct a panel data of 6 economic regions in Vietnam from 2005 to 2015. We employ the structural equation modelling (SEM) approach to quantify the causal effect of socio-economic status (SES), accessing to skilled birth attendance (SBA) and other relevant factors on the disparity of IMR across regions.

**Results:** SES, which is measured by 3 indicators – illiteracy rate (IR), poverty rate (PR) and income per capita – is the dominant factor causing regional inequalities of infant mortality, followed by the use of SBA. Among these indicators, the PR is the most important one causing the regional disparity of IMR and accessing to SBA. The total effect of SES on infant mortality disparity is 2.6 times as high as that of accessing skilled healthcare personnel.

**Conclusion:** Bridging the regional gap of using skilled health personnel would contribute to improving the infant mortality inequality in Vietnam. This inequality, however, is not significantly improved only with medical interventions but also with broader and more comprehensive socio-economic interventions at both national and regional levels. Our findings confirm that poverty reduction and growth strategies should be the main focus to boost medical interventions and improve IMR all over the country.

## Background

Vietnam achieved a significant reduction of infant mortality rate (IMR) over the period 1990-2015, from 44.1 per 1000 live births in 1990 to 14.7 in 2015. However, the decline does not take place equally in all regions of the country. The highest mortality rates are seen in mountainous and underprivileged regions at 24.8 per 1000 live births, which is nearly 3 times as high as the rate in the most affluent area at 8.6 per 1000 live births, and nearly 2 times the overall country’s IMR in 2015.^1^ The disparity between wealthy and disadvantaged areas tends to persist over time.

 Vietnam is divided into 6 economic regions, ie, Red River Delta, Northern Midlands and Mountainous areas, North Central and Central Coastal areas, Central Highlands, Southeast area, and Mekong River Delta. These 6 regions differ significantly in terms of demographics, speed and level of their socio-economic developments. The Southeast area with a high annual income per capita, a high literacy rate and a low poverty rate (PR), has the best socio-economic status (SES), followed by the Red River Delta. Meanwhile, the Northern Midlands and Mountainous area has the lowest SES compared to other regions.

In addition to SES, the proportion of births attended by skilled health personnel (so-called the skilled birth attendance, SBA) is one of the key factors affecting the IMR, thus a benchmark to assessing the progress towards the targets of reducing IMR in low- and middle-income countries (LMICs).^2^ SBA is critical medical services to women and their newborns during pregnancy, childbirth and the postpartum period, contributing to the reduction of preventable causes of neonatal death of newborn babies such as birth asphyxia and severe infections in Vietnam.^3^ This proportion of SBA is significantly different among economic regions in Vietnam. For instance, it is varying from 100% in the Red River Delta to only 91% in Northern Midlands and Mountainous areas and 94.8% in Central Highlands.^4^

The huge gap of IMR among different economic regions has drawn a great deal of attention from policy-makers, healthcare providers, international donors and researchers in Vietnam. Particularly, the Vietnamese government report on the achievements of Millennium Development Goals has highlighted medical interventions such as SBA, vaccinations and family planning as major contributors to reduce the IMR.^5^ In a number of earlier studies, SES has been identified as the determinant of the use of SBA in Vietnam.^6,7^ In separate studies, SES also shows a strong relationship with the IMR.^8,9^ However, to our knowledge, no research considers these factors’ linkages as a systematic structure that affects the inequality in SBA and IMR simultaneously across economic regions in Vietnam.

As the goals of Vietnamese government is to bridge the gap of IMR among economic regions to move forward to Sustainable Development Goals (SDGs) in 2030,^10^ there is a need to advance understandings on which factors are dominant, and how they contribute to this disparity. In particular, we need to examine the pathways through which the determinants of IMR such as SES, SBA, vaccinations and other relevant factors are interrelated in a system of causal relationships and at varying levels of magnitude. This study, using structural equation modelling (SEM) analysis, can shed lights on those pathways to infant mortality inequality over the period 2005-2015 in Vietnam.

 This study makes several contributions to the current literature and practice. First, it provides evidence of the determinants to reducing infant mortality inequality among regions of Vietnam. The findings would be of interest of policy-makers, healthcare providers and international organizations who are working hard in improving mother and child’s health and addressing health’s inequity in Vietnam. Second, it uses SEM to model a system of causal relationships among all related factors that would be applicable in other countries with similar socio-economic conditions. Finally, the case of Vietnam provides the World Health Organization (WHO) more evidence of the infant mortality disparity within countries, which in turn would inform WHO in setting guidelines, definitions and framework to support LMICs to obtain 2030 SDGs.

## Methods

###  Data Construction and Sources

In this study, we analyze data of 6 economic regions from 2005 to 2015 to identify the main factors affecting infant mortality across regions in Vietnam. In each economic region, we collected IMR indicators and medical interventions factors (SBA rate, vaccination rate), population growth (total fertility rate, TFR) and socio-economic factors (ie, PR, income per capita, and illiteracy rate [IR] as a basic level of education) other relevant indicators. All indicators are observed over 11 years. Therefore, the dataset contains a tally of 66 observations, The medical intervention indicators are collected from the annual Statistics Health Yearbooks of the Vietnam’s Ministry of Health (MoH).^4^ Meanwhile, the IMR, population growth and other socio-economic factors are sourced from Vietnam’s national statistical agency, so-called the General Statistics Office (GSO) of Vietnam which provides authentic official and nationally representative statistics on a wide range of economic, social, population and environmental issues. The IMR, population growth and other socio-economic factors are collected through household surveys of which samples are selected in the way to represent regions, provinces/cities and the entire country.^1^

###  Variables of Interest

We use the IMR of the 6 economic regions over 11 years as the dependent variable, and the others as the explanatory variables that include SES, the rate of non-fully vaccination, the proportion of SBA, and the TFR. The selection of explanatory variables is based on the literature and the availability of data in the Vietnamese context.^10^

SES has been well known as an important factor that determines the IMR in both LMIC and high-income countries.^11,12^ In this study, annual income per capita, PR and IR are used as proxies for SES. The income per capita captures the level of average income across regions. It however does not capture individual income, therefore, can mask disparities among individuals’ income. This indicator is estimated on a logarithmic scale because on such a scale, equal distances between income ranges represent equal percentage changes. For example, the distance between $500 and $1000 (a 100% change) is the same as the distance between $1000 and $2000 (a 100% change). The PR describes the distribution of low income across regions. The higher rate of poverty and illiteracy are expected to lead to a higher IMR.^13^ We believe that these SES variables are informative to roughly depict differences of regional socio-economic conditions and to examine its effect on IMR.

For the other explanatory variables, vaccinations and SBA have been reported as key medical interventions contributable to the decrease of infant mortality in Vietnam.^5^ In this study, we hypothesize that those interventions directly affect IMR.

The TFR is an element of population growth, reflecting both the causes and effects of economic and social developments. It also reflects the varying demand for infant professional healthcare. In response, skilled birth personnel need to adjust accordingly to the TFR. We, therefore, model the TFR as an input to the rate of SBA.^2,5^

The definition and measurement of our dependent and explanatory variables are presented in Table 1.

**Table 1:** Definition and Measurements of Variables

**Variables**	**Unit**	**Label**	**Measurements**	**Sources**
**Dependent**				
IMR	Permille ** (**per thousand)	IMR	The number of deaths under 1 year of age per 1000 live births	Vietnam GSO
**Explanatory**	
The rate of SBA	Percentage	SBA	The proportion of births delivered by skilled birth attendants	Vietnam MoH
The rate of non-fully vaccination	Percentage	NON_VAC	The rate of infants under 1 year is not fully vaccinated against 10 diseases	Vietnam MoH
TFR	Percentage	TFR	The average number of live births of a woman that would have during her whole life if during her reproductive period her age-specific fertility rates were similar to that observed in the reference period, usually the last 12 months prior to the survey	Vietnam GSO
The income per capita on a logarithmic scale	Million VND	LOG_INC	Total income of households in reference year divided by their headcounts	Vietnam GSO
PR	Percentage	PR	The proportion of the population living below the poverty lines calculated by the GSO and the World Bank based on consumption. Details of the methods of calculations can be found at World Bank documents ^14^	Vietnam GSO
Adult IR	Percentage	IR	The proportion of illiteracy persons aged over 15 over the population aged over 15	Vietnam GSO

Abbreviations: SBA, skilled birth attendance; TFR, total fertility rate; GSO, General Statistics Office; MoH, Ministry of Health; PR, poverty rate; IR, illiteracy rate; IMR, infant mortality rate.

###  Data Analysis Using Structural Equation Modelling

SEM is a multivariate statistical analysis technique that is used to construct and analyze structural relationships between measured variables and latent factors with a system of linear equations. This modelling technique allows us to track not only the direct effects but also the indirect ones of each variable to the dependent variable,^15^ in our study, ie, IMR. A pathway diagram, which is a flowchart showing variables interconnected with lines indicating causal flows to the dependent variable, present the structure and outcome of this analysis.

**Figure 1:**
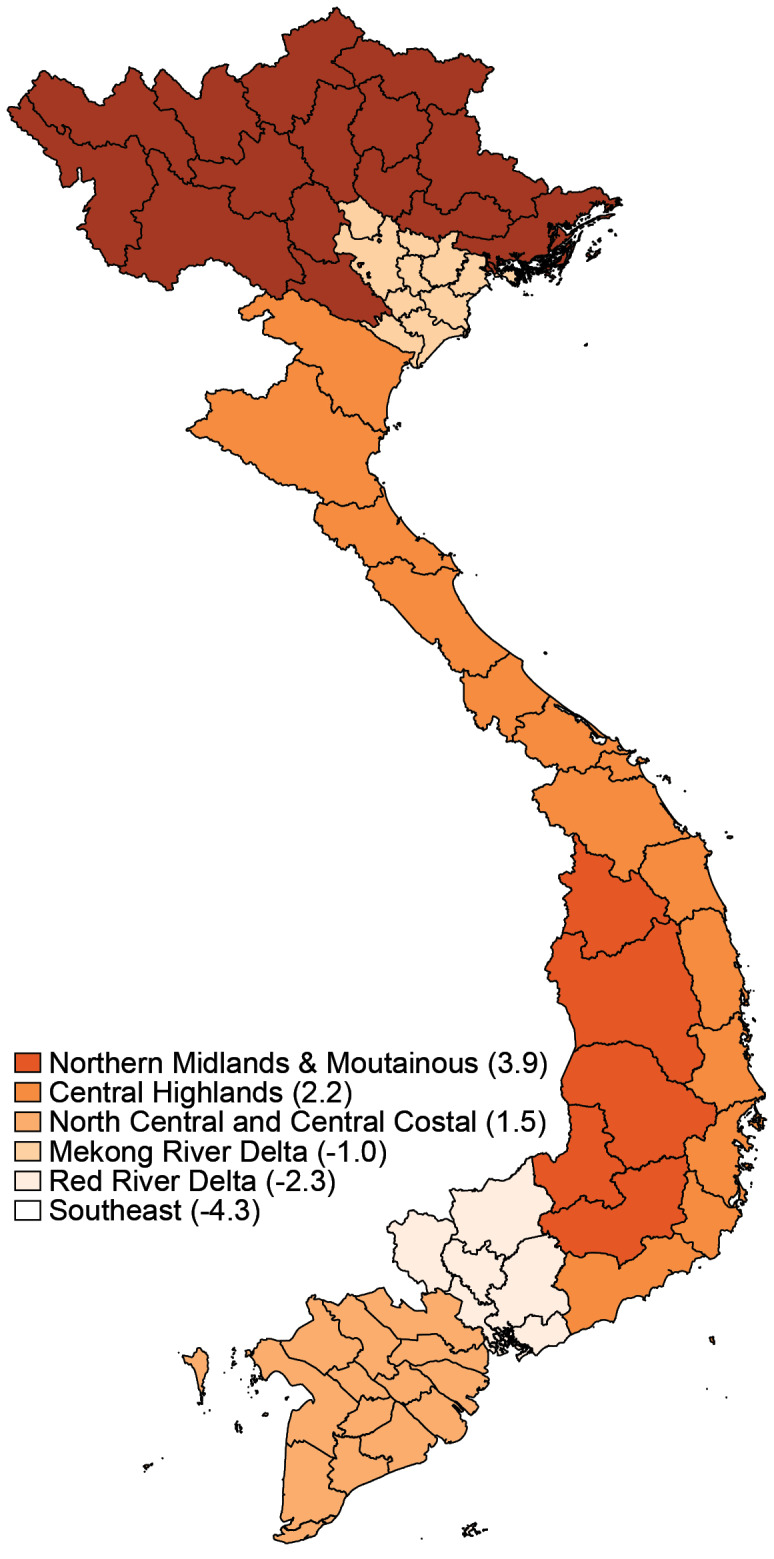
Six economic regions in Vietnam and their estimated average *SES_lat* during the period 2005-2015.

We hypothesize that besides the direct effect on IMR, the SES also affects the IMR through 2 indirect ways: the proportion of SBA and the non-fully vaccination rate. Those 2 latter variables, in turn, have direct effects on the IMR. As SES is not easily observable, we build a simple proxy for SES that is a common latent factor of 3 socio-economic measures, ie, IR, PR and log of income per capita. Hereinafter, we name this common latent factor as SES_lat. Figure 1 presented 6 economic regions in Vietnam and their estimated average SES_lat during the period 2005-2015.

The average *SES_lat* is simply the annual average of our estimated latent SES over the period 2005-2015 in each region. The higher is *SES_lat*, the higher rates of illiteracy and poverty are. Thus, the regions in darker (alerting) orange color are the ones with more socio-economic disadvantage. The map is built using GIS from the Database of Global Administrative Areas (GADM).

**Figure 2:**
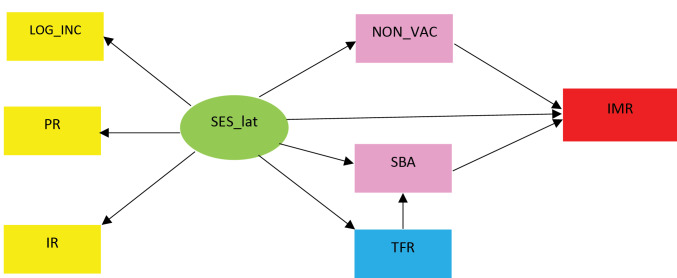
The Hypothesized Model of Inter-correlation Among SES, Medical Intervention, Population Growth, and IMR.

Abbreviations: SBA, skilled birth attendance; TFR, total fertility rate; PR, poverty rate; IR, illiteracy rate; IMR, infant mortality rate; SES, socio-economic status.

Figure 2 illustrates the hypothesized model demonstrating causal flows from our groups of explanatory variables (SES, medical intervention, and population growth) to the IMR. Lines with arrows represent the directional links among variables.

 To test this hypothesis, we first estimate the latent factor of SES across regions by applying a maximum likelihood factor analysis to 3 highly correlated variables, ie, IR, PR and log of per capita income, as what follows:

*IR = SES_lat+ε*_1_

*PR = α SES_lat + ε*_2_

*LOG_INC = β SES_lat + ε*_3_

As clearly seen, the common factor *SES_lat* is based on the *IR*, thus representing an unfavourable socio-economic indicator of each region. That means higher *SES_lat* implies a lower SES. As a result, *SES_lat* has a positive effect on *PR*, and negative effect on the log of income.

 We then use this estimated latent SES indicator to investigate its intertwined relationship with IMR and other factors. We start with a simple random effect model, where 2 separate linear relationships are estimated as follows:


$$\begin{array}{c} IMR_{i,t}=\alpha_{1}+b_{1}SBA_{i,t}+c_{1}NON_{VAC_{i,t}}+e_{1,i,t}\\ SBA_{i,t}=\alpha_{2}+b_{2}SES\_lat_{i,t}+c_{2}TFR_{i,t}+e_{2,i,t} \end{array}$$

where *i* and *t* indicate region *i* at year *t*, and (*e*_1_*, e*_2_) are residuals. In this set-up, SES affects IMR indirectly through the SBA channel. As our sample size is small, we use bootstrap with 100 replications to obtain the statistics for all estimated models.

This simple model shows that there is a significant impact of SES on SBA, as well as SBA on IMR. However, the Breusch-Pagan Lagrangian tests for random effect of these 2 equations indicate that the residuals may have different random effects across regions, thereby using this pooled OLS is not adequate (see Supplementary file 1 for more details). We, therefore, proceed to our SEM which can provide a better understanding.

 Our system of linear relationships in SEM is represented by

**Figure:**
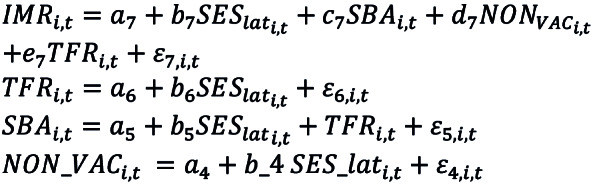


where (*ε*_4_*, ε*_5_*, ε*_6_*, ε*_7_) are iid random residuals. In this model, the latent variable, SES_lat, affects IMR not directly but indirectly through influencing vaccination rate, SBA, and TFR. Given our small sample size, we apply a bootstrap method to make statistical inference, which basically builds an empirical distribution for a statistic by resampling the available data appropriately. The indirect and direct effects of explanatory variables on the IMR are estimated, and the causal relationships between variables are graphed in a pathway diagram (see the result section for more details).

Specification tests: The goodness of fit of the 2 first random effect models are assessed by Breusch and Pagan Lagrangian test. The goodness of fit of SEM at overall and equation level is conducted to evaluate if the hypothesized model reflects the observed data and how much of the variance of explanatory variables is being explained by the model. We report 4 statistics of the model fitness – the model chi-square, comparative fit index (CFI), root mean square error of approximation (RMSEA) and standardized root mean square residual (SRMR).^16,17^

It should be noted that this study does not seek to infer to individuals’ preferences by using regional data, therefore, it is not subject to an ecological fallacy.^18^

## Results

###  Overview of IMR and SBA Over the Period 2005-2015

**Figure 3:**
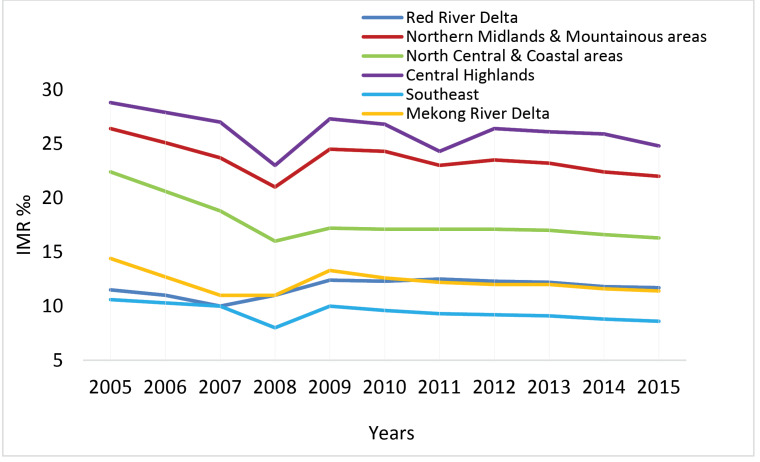
The Trend of IMR by Economic Regions in Vietnam. Abbreviation: IMR, infant mortality rate.

As shown in Figure 3, the regional IMR was gradually decreasing during the period 2005-2015. The disparity among economic regions, however, was still unchanged and huge, especially between low SES regions such as Central Highlands, Northern Midlands and Mountainous areas, and high SES areas such as Red River Delta and Southeast region. The biggest IMR gap during the time was nearly 17 ‰.

**Figure 4:**
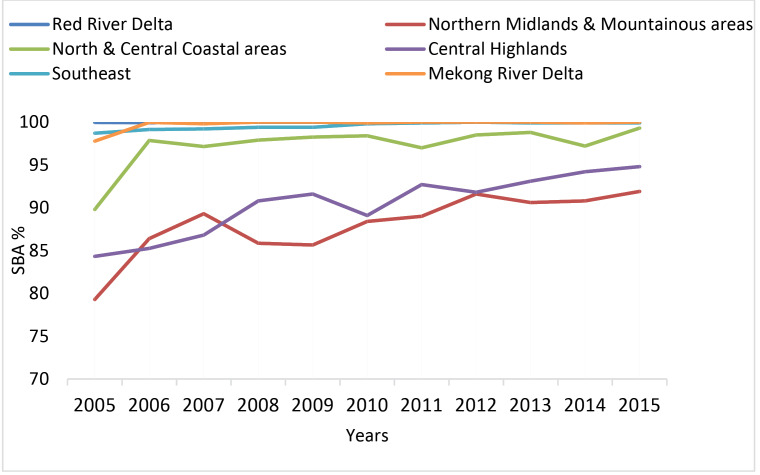
The Trend of SBA by Economic Regions in Vietnam. Abbreviation: SBA, skilled birth attendance.

Figure 4 demonstrates the upward trend of the proportion of SBA. The most interesting aspect of this trend is that the gap in the proportion of SBA has been gradually bridged among economic regions. However, the proportion of SBA has been still lower in Central Highlands, Northern Midlands and Mountainous areas, as compared to Red River Delta and Southeast regions.

###  Descriptive Statistics of Independent and Explanatory Variables by Regions, 2005-2015

Table 2 compares the summary statistics of IMR and explanatory variables among 6 economic regions. What stands out in the table is that Central Highlands and Northern Midlands & Mountainous areas are regions with the lowest socio-economic indicators and proportion of SBA; the highest TFR and IMR. In terms of immunization coverage, although Central Highlands has the highest non-fully coverage rate of 8.97%, this rate is not much different as compared with the Southeast region at 6.53%.

**Table 2:** Means and Standard Deviations of Independent and Explanatory Variables by Economic Regions in Vietnam

**Variable**	**Red River Delta**	**Northern Midlands & Mountainous Areas**	**North Central & Central Coastal areas**	**Central Highlands**	**Southeast**	**Mekong River Delta**
IMR	11.7 (0.78)	23.55 (1.51)	17.84 (1.98)	26.21 (1.67)	9.41 (0.78)	12.20 (1.02)
SBA	99.97 (0.04 )	88.07 (3.66)	97.29 (2.59)	90.41 (3.58)	99.57 (0.43)	99.58 (0.85)
NON_VAC	3.95 (5.55)	5.39 (2.48)	7.10 (6.68)	8.97 (10.59)	6.53 (4.00)	5.42 (2.98)
TFR	2.12 (0.08)	2.32 (0.16)	2.29 (0.06)	2.62 (0.25)	1.70 (0.10)	1.87 (0.07)
LOG_INC	2.42 (0.35)	1.87 (0.26)	2.00 (0.29)	2.07 (0.27)	2.8 (0.22)	2.21 (0.25)
PR	7.52 (2.83)	24.72 (4.36)	18.05 (4.67)	20.04 (5.02)	2.15 (1.15)	11.09 (2.50)
IR	2.96 (1.09)	12.69 (2.26)	6.20 (0.97)	10.89 (2.07)	4.49 (1.73)	8.50 (1.62)

Abbreviations: SBA, skilled birth attendance; TFR, total fertility rate; PR, poverty rate; IR, illiteracy rate; IMR, infant mortality rate. Note: The number in parentheses is the standard deviation.

###  Estimated Direct, Indirect and Total Effects of Explanatory Variables on IMR 

Table 3 illustrates the effect of income, PR and IR on *SES_lat*. An increase in this unfavorable *SES_lat* indicator triggers a 1% increase in IR, approximately 2.76% increase in the PR, and a 0.12% decrease in income.

Our estimated indirect and direct effects of explanatory variables on IMR are presented in Table 4. We find that 2 dominant factors contributing to reducing IMR during the period are SES (ie, SES, which is opposite to the *SES_lat*) and SBA. Of those, the total effect of SES on IMR is 2.6 times as high as the effect of SBA on IMR. This notably larger effect of SES on IMR is due to its magnified influence through the indirect pathway, reducing the TFR and the non-vaccination rate while boosting the SBA rate.

**Figure 5:**
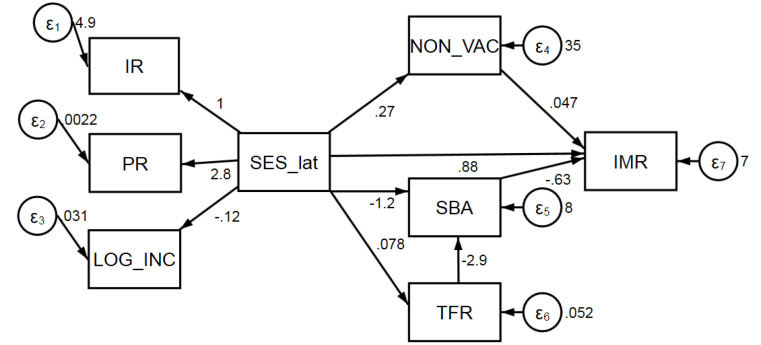
Pathway Diagram Representing the Major Drivers of Infant Mortality Inequality in Vietnam. Abbreviations: TFR, total fertility rate; PR, poverty rate; IR, illiteracy rate; IMR, infant mortality rate.

The inter-relationships among variables are graphed in Figure 5. The arrows show the directional and causal pathways among variables within the model. Numbers alongside the arrows represent the coefficients of causal variables to their dependent variables. The small circles with epsilon from 1-7 indicate unique error terms of the system that also randomly effect our variables of interest. For example, besides *SES_lat*, other random factors such as transportation condition and religious diversity also affect the use of SBA, and included in a random error *ε*_5_.

**Table 3:** Effect of Income, Poverty Rate and Illiteracy Rate on *SES_lat*

**Variable**	**Estimated Coefficient**	**Standard Error**	**95% CI**
IR	Reference		
LOG_INC	-0.12^**^	0.03	(-0.18, -0.06)
PR	2.76^**^	0.53	(1.71, 3.81)

Abbreviations: PR, poverty rate; IR, illiteracy rate. **^**^P < .01.

**Table 4:** Effect of Explanatory Variables on IMR

**Variable**	**Direct Effect**	**Indirect Effect**	**Total Effect**
IMR			
SES_lat	0.88**	0.77**	1.65**
SBA	-0.63**	NA	**-**0.63**
NON_VAC	0.05	NA	0.05
TFR	NA	1.85	1.85
SBA			
SES_lat	-1.44**	NA	-1.44**
TFR	-2.92*	NA	-2.92*
NON_VAC			
SES_lat	0.27	NA	0.27
TFR			
SES_lat	0.08**	NA	0.08*

Abbreviations: SBA, skilled birth attendance; TFR, total fertility rate; IMR, infant mortality rate. *^*^P < .05, **^**^P < .01.

The specification tests for the goodness of fit yields χ^2^= 32.09, *P* < .01; CFI = 0.90; SRMR = 0.04; RMSEA=0.32. All standardized residual co-variances are less than 0.37. Although the chi-square and RMSEA are not within the cut-off good fit (see Supplementary file 2 for more details) because of the small sample size of this study, the other indices, CFI and SRMR, show that the model fits well to the data.

 Overall, the results indicate that SES has causal relationships with IMR and SBA. The direct effect of SES on IMR is higher than the direct effect of SBA on IMR. In addition, SES also has an indirect effect on IMR through the SBA. Among the 3 indicators that measure the SES, the PR is the most important factor linked to the regional gap of SBA and IMR.

## Discussion

 Although a steady upward trend in SES and in the access of SBA, together with a downward trend in IMR has been observed in all economic regions in Vietnam, the regional gap of infant mortality remains unchanged. This study provides evidence to explain this infant mortality inequality. It also goes beyond simply demonstrating the linear relationship among IMR and its predictors to understand the pathway through which these predictors are interrelated in a set of linear and causal relationships.

SES is at the center of driving the disparity of IMR across the country. It has both indirect and direct effects on IMR. The direct effect on IMR is consistent with findings in a large number of studies and reviews that have highlighted SES as the critical determinant of infant mortality.^19-22^ The PR significantly affects the infant mortality inequality in Asia countries is also highlighted in the World Mortality Report 2015 published by the United Nations using national Health Demographic Surveys. Vietnam case, unfortunately, is not covered in this analysis. Therefore, this study strengthens the conclusions by adding up more evidence from Vietnam.^23^ Moreover, it further support the views of the indirect effect of SES on IMR through SBA is also similar to findings by Afulani and Moyer in Ghana.^24^

 Although all 3 SES indicators have direct and indirect effects on IMR, the PR has a stronger effect on the IMR and SBA than the income per capita and IR. This finding has an important implication for interventions that emphasizing the reduction of regional PR would be more likely to bridge the regional gap in IMR. Therefore, medical interventions should equally align with the implementation of the comprehensive poverty reduction and growth strategy across the country.

The proportion of SBA has a direct effect on the IMR, but it is also being affected by SES. This finding reflects 2 important issues of service users and providers in Vietnam. In terms of providers, the quantity, quality and distribution of skilled birth attendants have not been equally placed among economic regions.^25^ Given the regions with low SES have difficulties in attracting and retaining highly skilled health workers, the quality of SBA is not homogeneous among regions. Therefore, the variation of quality of SBA of regions should be taken into account since the competence of SBA is playing a critical role to the reduction of IMR.^26^ This appreciates the WHO’s effort to provide a new definition of SBA^2^ to address the disparity in quality of SBA. In terms of users, inequality of using maternal healthcare, including SBA is frequently due to contextual issues.^27^ In Vietnam, the income per capita, education and beliefs are most likely the barriers for an ethnic minority group of women, most of them live in Northern Midlands and Centre Highlands, to use birth support services. Moreover, language is also a barrier for midwives to reach out and support these ethnic women.^28^

Unlike other studies in LMICs,^29,30^ the full vaccination coverage does not statistically significant affect regional IMR in Vietnam. Furthermore, the SES is not statistically significantly associated with the vaccination coverage rate. Vietnamese contextual reasons would explain this finding. Firstly, the National Expanded Program of Immunization since 1981 has been successfully implemented. It does not just provide free vaccinations for infants in the whole country, but also especially focus on targeting infants living in disadvantaged areas.^31^ Secondly, the coverage rate is now substantially high in all regions; therefore, the community immunity or herd immunity – which give protection to a community where the disease cannot spread – has occurred.^32^ Lastly, many of the vaccinations are the first dose, and need following up in later years. Thus, vaccinations may have long term effects, and not reveal significantly in the first year of life.

 In conclusion, SES and SBA are the main factors affecting regional inequality of infant mortality in Vietnam. Closing the gap of SBA would contribute to improving the infant mortality inequality. More importantly, SES, in particularly the poverty, play a critical role in not only the inequality of IMR but also the regional disparity of accessing to SBA in Vietnam. Therefore, addressing this inequality cannot be done only within the healthcare system but in a broader socio-economic context. It requires more comprehensive interventions at national poverty reduction and growth strategy all over the country. The case of Vietnam raises an issue that infant mortality disparity within a country should be considered and factored in when setting and assessing the SDGs. This suggests WHO promote more evidence of the infant mortality disparity within countries which would inform WHO in setting guidelines and definitions and framework to support LMICs to obtain 2030 SDGs.

## Limitations

 The main limitation of this study is that it cannot account for the quality variation of SBA across regions of the country and how SES effects on the quality of SBA. This is an important question for further research. In addition, the small sample size of our dataset does not allow us to estimate the dynamic effects of IMR overtime on itself, ie, including IMR in previous years as additional explanatory variables in the SEM. The same kind of limit is applied to other variables. For instance, the impacts of SES, medical interventions, and population growth on IMR might last several years and at different magnitudes.

## Ethical issues

 This study used secondary data that have been published by Vietnam GSO and Vietnam’s MoH, Hanoi, Vietnam. The data did not cover any individuals and their personal health status.

## Competing interests

 Authors declare that they have no competing interests.

## Authors’ contributions

 MPN and CMN contributed equally to the paper’s design and writing.

## Authors’ affiliations

^1^Department of Medical Services Administration, Ministry of Health, Hanoi, Vietnam. ^2^Queensland University of Technology, Brisbane, QLD, Australia. ^3^School of Nursing, Indiana University, Bloomington, IN, USA.


Supplementary file 1. Goodness of Fit of the Random Effect Model.

Supplementary file 2. Goodness of Fit of the SEM.

## Key Messages

**Implications for policy makers**

The findings would be evidence-based for both the medical interventions and poverty reduction interventions at regional and national levels in Vietnam. They would be of interest of policy-makers, healthcare providers and international organizations who are working hard in improving mother and child’s health and addressing health’s inequity in Vietnam.

The case of Vietnam would provide the World Health Organization (WHO) more evidence of the infant mortality disparity within a country, which in turn would inform WHO in setting guidelines and definitions and framework to support low- and middle-income countries (LMICs) to obtain 2030 Sustainable Development Goals (SDGs).

**Implications for public**

The poverty rate (PR) in Asia countries is significantly affecting the infant mortality inequality and highlighted in the World Mortality Report 2015 by the United Nations, using national Health Demographic Surveys with no data of Vietnam. This study, therefore, contributes another evidence from Vietnam to strengthen the United Nations’ conclusions. More importantly, the poverty plays a critical role in not only the inequality of infant mortality rate (IMR) but also the regional disparity of accessing to skilled birth attendance (SBA) in Vietnam. Finally, the study employs structural equation modelling (SEM) to model a system of causal relationships among all related factors that would be applicable in other countries with similar socio-economic conditions.
