# Supplementary material for: Dominant Factors Affecting Regional Inequality of Infant Mortality in Vietnam: A Structural Equation Modelling Analysis
Source: Int J Health Policy Manag. 2020 Apr 29;10(8):475–82. doi: 10.34172/ijhpm.2020.59 (PMC9056204; doi:10.34172/ijhpm.2020.59)
Supplement: Supplementary file 1 — Goodness of Fit of the Random Effect Model. [file ijhpm-10-475-s001.pdf]

Supplementary file 1. Goodness of fit of the random effect model

Dependent variables	Explanatory variables	Estimated Coefficient	Bootstrap standard error	95% CI
IMR	SBA	-0.42	0.09**	(-0.58, -0.25)
	NOV_VAC	0.01	0.04	(-0.07, 0.09)
	Constant	56.77	8.19**	(40.72, 72.81)
	Chi-square		24.14**	
	Breusch-Pagan Lagrangian test		103.13**	
SBA	SES_lat	-1.12	0.20**	(-1.52, -0.72)
	TFR	-4.63	1.31**	(-7.19, -2.07)
	Constant	106.78	2.81**	(100.27,111.3)
	Chi-square		65.98**	
	Breusch-Pagan Lagrangian test		149.56**	

Abbreviations: SBA, skilled birth attendance; TFR, total fertility rate; IMR, infant mortality rate.

** $P < .01$.
