# Supplementary material for: Dominant Factors Affecting Regional Inequality of Infant Mortality in Vietnam: A Structural Equation Modelling Analysis
Source: Int J Health Policy Manag. 2020 Apr 29;10(8):475–82. doi: 10.34172/ijhpm.2020.59 (PMC9056204; doi:10.34172/ijhpm.2020.59)
Supplement: Supplementary file 2 — Goodness of Fit of the SEM. [file ijhpm-10-475-s002.pdf]

Supplementary file 2. Goodness of Fit of the SEM

1. **Overall goodness of fit**

Measure	Model estimation	Cut-off for good fit
Model Chi-Square	0.00	> 0.05
Comparative Fit Index (CFI)	0.90	≥0.90
Root Mean Square Error of Approximation (RMSEA)	0.38	<0.08
Root Mean Square Residual (SRMR)	0.05	<0.08

2. **Equation Level Goodness of fit**

**Variable**	**Variance**			**R-square**	**mc**	**mc2**
	**Fitted**	**Predicted**	**Residual**			
IMR	41.22	34.27	6.96	0.83	0.91	0.83
SBA	27.93	19.92	8.02	0.71	0.84	0.71
Overall				0.79		

mc: correlation between dependent variables an its prediction

mc2: the Bentler-Raykov squared multiple correlation coefficient

3. **Residuals of observed variables**

   Mean residuals

	IMR	SBA	NON_VAC	TFR	SES_lat
IMR	-0.007	-0.000	-0.000	-0.000	0.000

Covariance residuals

	IMR	SBA	NON_VAC	TFR	SES_lat
IMR	-0.007				
SBA	0.006	-0.000			
NON_VAC	-0.076	0.120	0.000		
TFR	0.366	0.000	0.061	-0.000	
SES_lat	-0.000	0.000	0.000	-0.000	0.000

Abbreviations: SBA, skilled birth attendance; TFR, total fertility rate; IMR, infant mortality rate.
